# Mechanisms and implications of bacterial–fungal competition for soil resources

**DOI:** 10.1093/ismejo/wrae073

**Published:** 2024-05-01

**Authors:** Chaoqun Wang, Yakov Kuzyakov

**Affiliations:** National Key Laboratory of Wheat Improvement, College of Agronomy, Shandong Agricultural University, Tai'an 271018, Shandong, China; Biogeochemistry of Agroecosystems, University of Göttingen, Göttingen 37077, Germany; Faculty of Land and Food Systems, The University of British Columbia, Vancouver V6T1Z4, Canada; National Key Laboratory of Wheat Improvement, College of Agronomy, Shandong Agricultural University, Tai'an 271018, Shandong, China; Department of Soil Science of Temperate Ecosystems, University of Göttingen, Göttingen 37077, Germany

**Keywords:** carbon and energy availability, carbon and energy fluxes, exploitative competition, interference competition, microbial community, soil organic matter

## Abstract

Elucidating complex interactions between bacteria and fungi that determine microbial community structure, composition, and functions in soil, as well as regulate carbon (C) and nutrient fluxes, is crucial to understand biogeochemical cycles. Among the various interactions, competition for resources is the main factor determining the adaptation and niche differentiation between these two big microbial groups in soil. This is because C and energy limitations for microbial growth are a rule rather than an exception. Here, we review the C and energy demands of bacteria and fungi—the two major kingdoms in soil—the mechanisms of their competition for these and other resources, leading to niche differentiation, and the global change impacts on this competition. The normalized microbial utilization preference showed that bacteria are 1.4–5 times more efficient in the uptake of simple organic compounds as substrates, whereas fungi are 1.1–4.1 times more effective in utilizing complex compounds. Accordingly, bacteria strongly outcompete fungi for simple substrates, while fungi take advantage of complex compounds. Bacteria also compete with fungi for the products released during the degradation of complex substrates. Based on these specifics, we differentiated spatial, temporal, and chemical niches for these two groups in soil. The competition will increase under the main five global changes including elevated CO_2_, N deposition, soil acidification, global warming, and drought. Elevated CO_2_, N deposition, and warming increase bacterial dominance, whereas soil acidification and drought increase fungal competitiveness.

## Introduction

Bacteria and fungi are by far the key living components in soils in terms of biodiversity, biomass, and their impacts on biogeo-chemical processes [1]. They always coexist with each other in soils and form complex interactions [2, 3] that are crucial for their survival, adaptation, establishment, maintenance, and functions [4]. These ubiquitous interactions can be classified within classical ecological theory as mutualism (a win–win interaction), competition (a loss–loss interaction), commensalism (a win–neutral interaction), parasitism (a win–loss interaction), amensalism (a loss–neutral interaction), and neutralism (a neutral–neutral interaction) (Glossary Box). Among these interaction types, competition for resources dominates these interactions in soils [5, 6]. Consequently, microbial adaptation predominantly involves competitive success [7]. This is because competition for limited space and resources (e.g. carbon [C] and energy sources, nutrients, water) is pervasive [8, 9]. Competition is also an important mechanism to increase microbial community stability by harboring various metabolically redundant species [10] and restricting microbial pathogen overgrowth [11]. In addition to its impact on microbial community structure, bacterial–fungal competition critically regulates multiple ecosystem functions such as nutrient cycling; decomposition of litter, rhizodeposits, and soil organic matter (SOM); soil structure formation’ increase in soil fertility; suppression of plant diseases; support of plant productivity; and enhancement of the resistance and resilience of ecosystems [12–14]. Despite growing awareness that bacteria and fungi have immense capacities to affect global biogeochemistry and multiple ecosystem functions, their competition is frequently overlooked in soil microbiome studies.

Competition for C and energy is stronger than for other resources because of the large overlap between bacterial and fungal demands for organic compounds and nutrients [15–17] and for the same soil locations (habitats) [15]. Importantly, the competition for energy—mostly stored in organic compounds photoassimilated by plants—may be far stronger than that for other resources. This is supported by the fact that microbial C use efficiency (CUE) in soil is commonly <0.4 [16, 17], indicating that >60% of C is used to obtain energy by oxidation of organic compounds to CO_2_ but not for structural C. Moreover, microorganisms use considerable C amounts to synthesize energy storage substances [18], which are also accounted by CUE estimations. Many microbial processes require energy but no C investment, resulting in a lower microbial energy use efficiency (EUE) than CUE [19, 20]. Within competitive interactions, both groups must always allocate energy to obtain the most limiting resource and to overcome the negative effects of competition [21]. This makes understanding the mechanisms controlling bacterial–fungal competition for C and energy sources crucial for untangling biogeochemical processes of C cycling and stabilization.

Microbial competition encompasses two main types: “exploitative competition” (also termed scramble competition, Glossary Box), in which one population rapidly consumes the limiting resource required by another without direct interactions between two populations, and “interference competition” (also termed contest competition, Glossary Box), in which competitive populations generate direct antagonistic interactions, with one population appropriating the resource by competitive success [7, 22]. Bacteria and fungi have developed various mechanisms of exploitative and interference competition with each other (see section Mechanisms of bacterial–fungal competition below), regulating the ecological niche differentiation between these two major kingdoms [5, 23, 24]. Bacteria are usually characterized by fast uptakes of labile C and energy resources [25], whereas fungi efficiently use more recalcitrant C sources [26, 27]. Fungi can expand their spatial niches to forage C and nutrient resources by forming hyphae and mycelium [28].

Various biotic factors (e.g. plant species, root–microbial interactions, microbial diversity and density) and abiotic limitations (e.g. the availability of C, energy, and nutrients, pH, moisture, aeration, temperature) can influence the competition in soils [4, 29]. Various global change processes (e.g. elevated CO_2_, nitrogen deposition, soil acidification, warming, drought) strongly affect these biotic and abiotic factors, thereby regulating the competition. For example, plant growth and fresh C input (e.g. “rhizodeposits” and “root exudates”; Glossary Box) from plants into soil commonly increase under elevated CO_2_ [30], which may reduce the intensity of bacterial–fungal competition for C and energy [19]. The responses to global change are kingdom-specific because bacteria and fungi have contrasting nutrient demands (e.g. C:N:P stoichiometries) and sensitivities to temperature, pH, moisture, and oxygen concentration. Thus, even a slight change in the biogeochemical environment may lead to a strong impact on microbial metabolism and demand for C and energy. Accordingly, global change strongly affects competitiveness. Considering that the stability and functioning of ecosystems depend strongly on the performance and balance of bacteria and fungi, understanding and predicting the response of such competition to global change is one of the most pressing research questions. What we urgently need to determine is how bacterial–fungal competition for C and energy and its impacts on biogeochemical processes change under changing climate. This, in turn, will benefit protecting, managing, and mitigating ecosystem resistance and resilience.

Heterotrophic respiration by SOC decomposition has globally increased as a result of climate change, thus contributing to increased atmospheric inputs of CO_2_. However, losses of soil C to the atmosphere could be countered by increased soil C inputs due to increased plant growth and autotrophic fixation by soil microorganisms. Also, the temperature sensitivity of SOC decomposition depends on the quantity and chemistry of plant litter and pre-existing SOC. Thus, even within specific biomes, the local biogeochemical environment strongly influences microbial metabolic responses to climate.

In this review, we assess the demands and preferences of bacteria and fungi for C sources in soil. We then summarize the competition mechanisms and resulting niche differentiation. Finally, we outline the effects of various global changes on bacterial–fungal competition for C and energy resources under real soil conditions.

**Glossary Box**

**Amensalism:** A relationship between organisms of two species, in which one is suppressed or destroyed and the other is unaffected.

**Commensalism:** A relationship between organisms of two species in which one organism (commensal) benefits while the other organism (host) of the association is neither benefited nor harmed.

**Competition:** A relationship between organisms of two species in which both organisms compete for the same resources within an environment at the same time.

**Cross-feeding:** An interaction between organisms of two or more species in which metabolic products of one organism are utilized by the other(s).

**Energy availability:** The ratio of the energy obtained to energy consumed through any activity to the energy that a (micro)organism or community must invest to utilize an organic compound under real soil conditions.

**Exploitative competition:** Competition in which one population consumes the resources required by another without direct contact between the two populations.

**Interference competition:** Competition in which one population suppresses or stops the growth of another by secreting harmful products.

**Microbial necromass:** Microbial residues—the remains of dead microbial cells, cell fragments, cell organelles, and cytoplasm.

**Mutualism:** A relationship in which each organism in interaction gets benefits from the association.

**Neutralism:** A relationship in which both organisms are not affected with respect to their survival and growth.

**Niche:** A multidimensional abstract space of resources and abiotic and biotic conditions enabling the species to maintain a viable population.

**Niche differentiation:** The process by which competing organisms use the environment differently to decrease the competition. Spatial, temporal, and chemical niche differentiations are common.

**Parasitism:** A relationship in which one organism (parasite) benefits and derives its nutrition from another organism (host), which is harmed.

**Priming effect:** A short-term change in SOM decomposition induced by pulses or continuous inputs of organic substances or nutrients to the soil.

**Rhizodeposits:** All compounds released by living roots through rhizodeposition.

**Rhizosphere:** Soil volume affected biochemically and physically by plant roots.

**Root exudates:** A part of rhizodeposits consisting of organic compounds passively released (lost) by living roots in the rhizosphere.

### Carbon and energy demands of bacteria and fungi

#### Carbon and energy sources of bacteria and fungi

All processes during bacterial and fungal growth, maintenance, and dormancy consume energy, and most of them consume C [19, 20]. Each group has preferences for organic compounds depending on their structural complexity and surface properties [31] and partly on metabolic specifics. Both bacteria and fungi can utilize various organic compounds to gain C and energy for their growth and maintenance in soil. According to the origin, the main C and energy sources can be classified as “rhizodeposits” (Glossary Box), plant litter, microbial necromass, organic fertilizers, and SOM (Table 1).

**Table 1:** Carbon and energy sources of bacteria and fungi in soil.

Groups	Compositions	Hydrophobicity	Dominant use group	Microbial succession	
				Early	Late
Rhizodeposits	Carboxylic acids	−	Bacteria	/	/
	Sugars	−	Bacteria	/	/
	Amino acids	−	Bacteria	/	/
	Phenolics	−/+	Fungi	Fungi	Bacteria
	Fatty acids	+	Fungi	Fungi	Bacteria
	Vitamins	−/+	Fungi	Fungi	Bacteria
	Purines	−	Bacteria	/	/
	Sterols	+	Fungi	Fungi	Bacteria
	Flavanones and nucleotides	−	Bacteria	/	/
	Enzymes	−	Fungi	Fungi	Bacteria
	Mucilages	−	Fungi	Fungi	Bacteria
	Root border cells	+	Fungi	Fungi	Bacteria
	Dead roots	+	Fungi	Bacteria	Fungi
	Sloughed root cells and root hairs	+	Fungi	Bacteria	Fungi
	Lysates	−	Fungi	Fungi	Bacteria
Plant litter		+	Fungi	Bacteria	Fungi
Microbial necromass		+	Fungi	Fungi	Bacteria
Organic fertilizers		+	Fungi	Bacteria	Fungi
Soil organic matter		−/+	Fungi	Bacteria	Fungi

Note: We used − and + if organic compounds are hydrophilic and hydrophobic compounds, respectively. “−/+” represented the group contains amphipathic organic compounds. We used / to indicate the direct uptake of compounds by microorganisms.

Estimates for the allocation of plant C to rhizodeposits range between 10% and 50% of photoassimilated C [32, 33], which is equivalent to 800–4500 kg C ha^−1^ year^−1^ in perennial and annual plants [34, 35]. Importantly, rhizodeposits include root exudates: various soluble, low-molecular-weight compounds, especially sugars, carboxylic acids, and amino acids [36, 37]. They are important C and energy sources because they are soluble and thus very easily available for bacteria and fungi, and therefore, they require virtually no additional costs for dissolution and uptake. Further, many of these compounds are the key substances in metabolic cycles.

Microbial utilization of plant litter depends on the decomposition stage. At the early stage, soluble and labile molecules (e.g. sugars, amino acids), leached from the cells broken by various processes, will be rapidly taken up by bacteria and fungi [38]. At the later litter decomposition stage, the remaining complex and recalcitrant compounds such as lignin, tannin, and chitin require high energy and C input for exoenzyme production necessary for hydrolysis and oxidation [39]. Microbial necromass C accounts for 35%–51% of total topsoil organic C [40], potentially serving as an important C and energy source [40, 41]. The depolymerization and decomposition of microbial necromass may be faster than that of complex compounds (e.g. lignin) in plant residues because the organic compounds are smaller and have a much higher C:N ratio (dominance of proteins and amino sugars) [41].

The energetic potential (e.g. Gibbs free energy [$\Delta{G}^o$]) of a given organic compound is reflected in the nominal oxidation state of all C atoms (NOSC) [42] and can be estimated using the following equation [19, 43]:


(1) \begin{equation*} \varDelta{G}^o=108\kern0.5em NOSC\hbox{--} 454 \end{equation*}

where Δ*Go* is the energy content (enthalpy) in organic compounds (potentially available for microorganisms) and NOSC is the nominal oxidation state of all C atoms in those compounds; the latter can be calculated using the following equation:


(2) \begin{equation*} NOSC=4-\frac{-Z+4C+H-3N-2O+5P-2S}{C} \end{equation*}

where *C*, *H*, *N*, *O*, *P*, and *S* are the stoichiometric values of the elements and *Z* is the net charge of the organic compounds.

**Figure 1:**
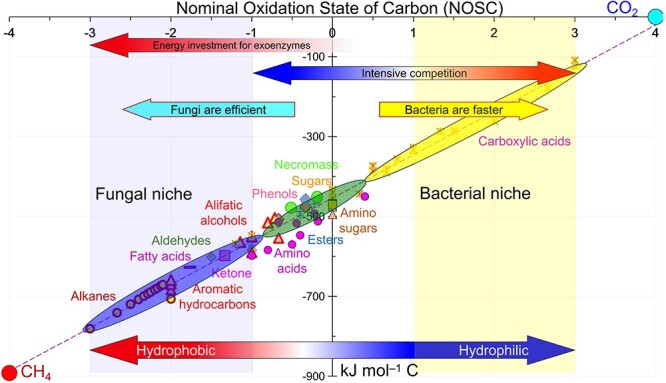
Conceptual representation of the nominal oxidation state of carbon (NOSC) (*x*-axis) and energy content (combustion enthalpy) (*y*-axis) in the main compounds as carbon and energy sources for microorganisms in soil.

The NOSC values of the main C and energy sources in soil increase in the following order: lipids, microbial necromass, lignin, amino acids, phenolics, plant litter, NaOH-extractable SOM, sugars, HCl-extractable SOM, and carboxylic acids (Fig. 1) [42, 43]. Even though the energy content in organic compounds decreases with increasing NOSC values, the energy availability (Glossary Box) increases. Microorganisms therefore preferentially utilize organic compounds with higher NOSC values.

### Hydrophobicity of carbon and energy sources

Generally, bacteria rapidly take up labile organic compounds, whereas fungi are often associated with recalcitrant forms [44, 45]. Besides many simple compounds (glucose, glycine, acetic acid), some complex compounds (starch) are predominantly taken up by bacteria [46]. Similarly, fungi assimilated up to two times more C from *N*-acetylglucosamine utilization than bacteria in the birch–willow system with a fungal: bacterial PLFA ratio of only 0.14 [47].

Microbial C utilization strongly depends not only on the structural complexity and energy availability but also on the solubility of organic compounds. This is because solubility determines the accessibility of organic compounds to microorganisms [27]. Soluble organic compounds diffuse through solution, enabling microorganisms to capture them from a larger soil volume. In contrast, insoluble compounds are commonly high-molecular-weight compounds or adsorbed on metal oxides, clay minerals, or organic matter [48], making them less available for microbial uptake. Soluble compounds are more effectively hydrolyzed or oxidized by exoenzymes compared to insoluble compounds [49], leading to strong competition between bacteria and fungi, especially for soluble compounds. Microorganisms must allocate considerable energy to break down the complex chemical (e.g. ligand exchange) and/or physical (e.g. electrostatic attraction, hydrophobic partitioning) associations between insoluble organic compounds and soil minerals [50]. This strongly decreases microbial CUEs and EUEs using insoluble organic compounds as substrates. Fungi have higher capacities to transform energy from organic compounds into their biomass than bacteria [19], which may increase their competitiveness for utilizing insoluble compounds.

Organic compounds can be differentiated into hydrophilic and hydrophobic compounds based both on structural complexity and surface hydrophobicity. Hydrophobic partitioning of organic compounds with hydrophobic moieties and functional groups (e.g. −OCH_3_, −CH_3_, −CN) from soil solution onto minerals counteracts microbial decomposition [48]. The higher the hydrophobicity of organic compounds, the slower their microbial decomposition [27]. Both bacteria and fungi therefore preferentially uptake and utilize hydrophilic organic compounds as their C and energy sources. The morphological (e.g. simple cell structure, large area-to-volume ratio) and physiological (e.g. fast growth and substrate uptake rates) features of bacteria (Table 2) help them to rapidly utilize hydrophilic compounds. Fungi have a higher hydrophobicity and more effective exoenzymes than bacteria (Table 2), enabling them to utilize hydrophobic compounds by oxidation and hydrolysis.

**Table 2:** Main differences in physiological and metabolic traits adapted by bacteria and fungi in soil.

Microbial traits		Bacteria	Fungi	References
Morphology	Cell type	Prokaryotic	Eukaryotic	*
	Cell number	Unicellular	Multicellular	
	Cell shape	Round, spiral, rod	Mainly filamentous	
	Cell size	0.5–5 μm	2–10 μm	
	Cell structure	Simple	Complex	
	Cell wall	Peptidoglycan	Chitin	
	Cytoskeleton	Absent	Microtubules or microfilaments	
	Area-to-volume ratio	High	Low	
	Hydrophobicity	Low	High	
Physiology	Maximal growth rate	0.3–1 h	12–24 h	*
	C:N ratio	4.8	8.7	[19]
	C:H ratio	0.57	0.56	[19]
	C:O ratio	2.2	2	[19]
	C:P ratio	42	42	[19]
	C:S ratio	333	333	[19]
	Filamentous growth	Only few groups	Common	*
	pH range niche	Narrow	Broad	*
	Oxidation state (NOSC)	−0.33	−0.53	[19]
	Biomass turnover rate	0.75–133 days	30–440 days	[51]
	Substrate uptake rate	Fast	Slow	[26]
	Respiration	Anaerobic and aerobic	Aerobic	*
	Water demand	High	Low	[52, 53]
	Maintenance energy	High	Low	[54]
	Sensitivity to disturbance	Low	High	[55]
	(Chemical and temporal) Niche width	Narrow	Broad	[27, 56–59]
Metabolism	C and energy sources	Inorganic and organic matter	Organic matter	*
	Nutrition	Autotrophs or heterotrophs	Heterotrophs	*
	Hydrolytic enzyme efficiency	Low	High	[60–63]
	Oxidative enzyme efficiency	Absent	High	[60–63]
	Enzyme diversity per species	Low	High	[60–63]
	Carbon use efficiency	Low	High	[19]
	Energy use efficiency	Low	High	[19]
	Maintenance	High	Low	[54]
	Biomass turnover	Fast	Slow	[51]

Note: Asterisks indicate that information on the differences between bacteria and fungi was obtained from https://microbenotes.com/bacteria-vs-fungi/.

### Preferences for carbon and energy sources

To evaluate the preferences for C and energy sources, we collected 155 data (see details in Supplementary materials; Table S1) on the incorporation of ^13^C-labeled substances into microbial biomarkers—phospholipid fatty acids (PLFAs). We designed Equation (3) to normalize ^13^C-enrichment in fungal relative to bacterial PLFAs (^13^C_Fungi_/^13^C_Bacteria_) to the biomass C content in fungi relative to bacteria:


(3) \begin{equation*} Microbial\ utilization\ preference\ (MUP)=\frac{\frac{{}^{13}{C}_{Fungi}}{{}^{13}{C}_{Bacteria}}}{\frac{Total\ {C}_{Fungi}}{Total\ {C}_{Bacteria}}} \end{equation*}

where ^13^C_Fungi_ and ^13^C_Bacteria_ are ^13^C enrichment in fungal and bacterial PLFAs, respectively, and (Total C_Fungi_) and (Total C_Bacteria_) are the C content in fungal and bacterial PLFAs, respectively. Higher MUPs indicate higher substrate-C assimilation by fungi versus bacteria. At MUP values higher than 1, fungi outcompete bacteria for the given substrate and vice versa.

**Figure 2:**
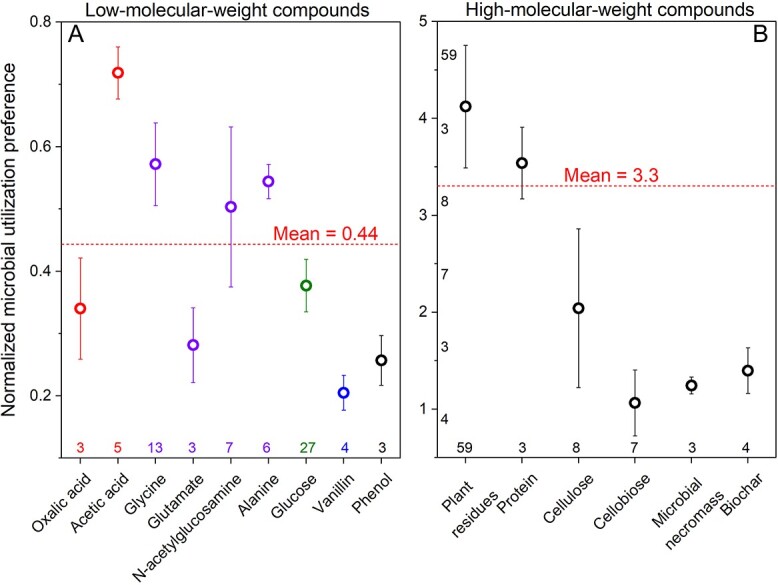
Normalized microbial utilization preference (MUP) using various simple (A) and complex (B) compounds as substrates incorporated into fungal PLFAs relative to bacterial PLFAs. Red dashed lines represent mean MUP values using simple (A) and complex (B) compounds as substrates. The closer the MUP value is to 1, the more similar the competitiveness of bacteria and fungi for the substrate. The data are means ± standard errors. Numbers on left show the number of studies. Note that the mean MUP preference of 0.44 for low-molecular-weight compounds (left) corresponds to 2.3 times stronger competitivity of bacteria than fungi.

The MUP values of 0.20–0.72 correspond to a bacterial competitiveness of 1.4–5 times stronger than fungi for small (MW < 200 Da) and hydrophilic compounds (Fig. 2A). In contrast, the MUP values for complex substrates (e.g. plant residues, microbial necromass, proteins, cellulose, cellobiose, biochar) ranged from 1.1 to 4.1 (Fig. 2B). Accordingly, bacteria outcompete fungi for simple substrates, while fungi have a major advantage regarding complex substrates.

**Figure 3:**
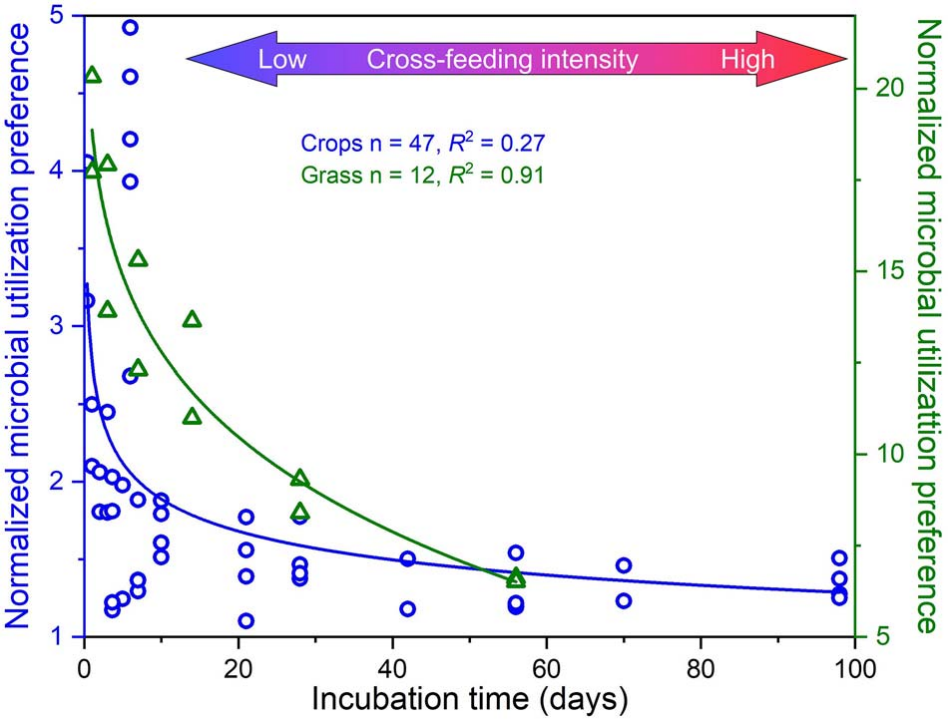
Relationships between normalized MUP using residues of crops (cycles) and grasses (triangles) as substrates, from which C was incorporated into fungal PLFAs relative to bacterial PLFAs and the incubation time (i.e. residue decomposition time). Relationships were determined by power regression analysis (both *P* < .001). The sharp decrease in MUP values with residue decomposition indicates the cross-feeding interactions (starting after 10–20 days) between bacteria and fungi.

**Figure 4:**
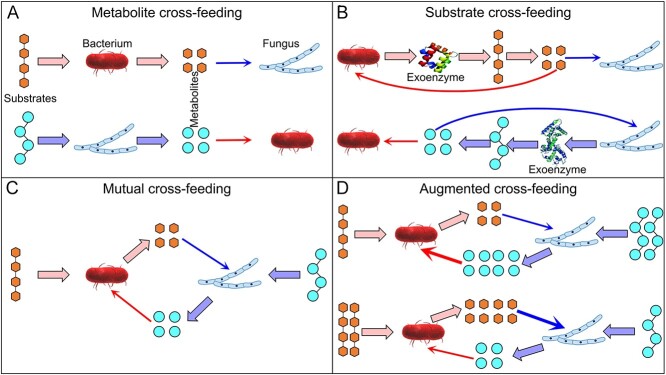
Bacterial–fungal cross-feeding types. (A) Metabolite cross-feeding: one organism feeds on a complex compound, releasing a waste metabolite that is used by another organism. (B) Substrate cross-feeding: one organism secretes exoenzymes to decompose complex compounds to simple compounds that are used by another organism. (C) Mutual cross-feeding: any combination of metabolite and substrate cross-feeding, in which bacteria and fungi are cross feeders. (D) Augmented cross-feeding: a subset of mutual cross-feeding, in which one organism supplies more of a cross-fed compound to another organism.

A gradual decrease in the MUP using plant residues as substrates (Fig. 3) with decomposition duration suggests that fungi are better competitors for such residues, especially at the early decomposition stage. Later, however, bacteria obtain more products directly or indirectly from fungi, reflecting the common phenomena of cross-feeding (Fig. 4). This is because bacteria can only utilize smaller compounds produced by plant residue decomposition, which takes time to occur [64]. This is also supported by the lower MUP for cellulose and cellobiose used as substrates (with fast degradation rates) than those using slow-degrading proteins and plant residues (Fig. 3).

Cross-feeding commonly assumes the utilization of metabolic products (organic compounds and nutrients) of one organism by another (Box 1), whereby only intracellular metabolism is commonly considered. Cross-feeding in soil, however, includes and probably mainly involves one microbial group utilizing the products released by extracellular reactions of exoenzymes produced by another group. Considering the omnidirectional losses of reaction products of exoenzymes by diffusion, we assume that the most cross-feeding in soil involves by mechanism 2: substrate cross-feeding (Fig. 4B). Although cross-feeding interactions are most likely positive, they create competition between organisms. Substrate cross-feeding is the basis for exploitative competition between organisms for products [65]. Although bacteria and fungi do not compete for products in other cross-feeding interactions (Fig. 4A, C, and D), they do compete to some extent for other shared resources (e.g. water, oxygen, nutrients) [7].

## Bacterial–fungal competition and niche differentiation

Limited C and energy availability in soils are the rule rather than the exception, therefore typically restricting the growth of all nonfilamentous bacteria, actinobacteria, and fungi [8, 66]. These limitations in soil cause bacteria to continuously compete with fungi for organic C and energy.

The competition for energy is much stronger than for C per se. Firstly, microorganisms can recycle C both intracellularly and extracellularly, which requires energy investment but not new C [19, 20]. Secondly, microorganisms must invest energy to reduce organic and inorganic compounds, which takes place without C utilization [19, 20]. Thirdly, microorganisms allocate substantial energy for maintenance [67], and various processes (e.g. cell division, metabolic shifts, cell motility, regulation of gene expression, energy spilling reactions) consume energy but no or little C [19, 20]. For example, the maintenance energy of bacteria (12 kJ mol^−1^ C h^−1^) is 50% higher than that of fungi (8 kJ mol^−1^ C h^−1^) under aerobic conditions at 30°C [54]. Both groups, however, require more energy to compete with each other, thus reducing the energy available for other functions [21]. This, in turn, increases the intensity of competition for energy.

Bacteria and fungi strongly compete for easily available C and energy sources, but they have also evolved both competitive and mutualistic strategies for decomposing recalcitrant organic compounds [24]. Gram-negative bacteria generally are fast-growing r-strategists that can rapidly uptake easily available substrates, whereas fungi and Gram-positive are slow-growing K-strategists that can efficiently utilize recalcitrant organic compounds [26]. This is reflected by much higher numbers of bacteria, especially Gram-negative strains, than of fungi in the rhizosphere, where roots continuously exude simple substrates [68]. The abundance of Gram-negative bacteria increases when glucose is added to soils, whereas fungal and Gram-positive bacterial abundances increase with the addition of recalcitrant organic matter [69]. The fungi-to-bacteria ratio always decreases after adding easily available C sources (e.g. sugars, amino acids) but increases in response to recalcitrant organic matter [69].

The two groups coexist in the many niches in soil (Table 3), increasing their competition for C and energy. Niche differentiation effectively reduces such competition. The winner of niche differentiation depends on their morphological (e.g. cell size, structure, hydrophobicity), physiological (e.g. growth rate, water and nutrient demands), and metabolic (e.g. enzymatic catalytic efficiency, C and energy use efficiency) properties (Table 2). The physico-chemical conditions (e.g. the complexity of organic compounds, O_2_ concentration, pH) of the niche also play a role.

**Table 3:** Chemical, spatial and temporal niche differentiation between bacteria and fungi.

Niche differentiation		Outcompeting kingdom	References
Chemical	Low-molecular-weight compounds	Bacteria	This paper
	High-molecular-weight compounds	Fungi	This paper
	Hydrophilic compounds	Bacteria	[43, 44, 70–72]
	Hydrophobic compounds	Fungi	[70]
Spatial	Rhizosphere	Bacteria	[73, 74]
	Detritusphere	Fungi	[75]
	Bulk soil	Bacteria	[76]
	Biopores	Fungi	[77]
	O horizon	Fungi	[75]
	Topsoil	Bacteria	[78, 79]
	Subsoil	Bacteria	[78, 79]
	Macroaggregates	Bacteria	[80, 81]
	Microaggregates	Fungi	[81]
	Small pores	Bacteria	[82, 83]
	Large pores	Bacteria	[82, 83]
	Oxic locations	Bacteria	[82]
	Anoxic locations	Bacteria	[82]
	Dead bodies of animals	Fungi	[3]
	Surfaces of organic matter	Fungi	[3]
	Surfaces of clay minerals and Fe oxides	Bacteria	[3]
Temporal	Earlier stage of litter decomposition	Bacteria	[38, 70, 84, 85]
	Later stage of litter decomposition	Fungi	[38, 70, 84, 85]

### Mechanisms of bacterial–fungal competition

**Figure 5:**
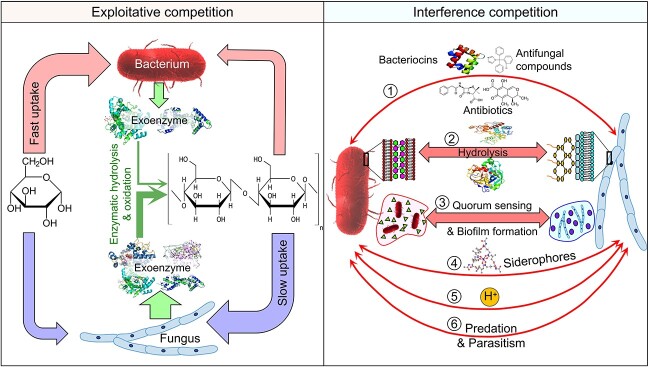
Strategies used by bacteria and fungi to compete for carbon and energy sources in exploitative (left) and interference (right) competitions. In exploitative competition, bacteria rapidly take up easily available compounds, thus outcompeting fungi, while the latter outcompete the former for complex compounds by relying on their diverse enzymes with high catalytic efficiency. Mechanisms adopted to outcompete each other in the interference competition from top to bottom include: (i) producing various compounds to suppress or kill each other, (ii) secreting specific hydrolytic enzymes (e.g. chitinases, proteases) to break down the cell walls or proteins of their competitors, (iii) producing siderophores to chelate iron (and other multivalent cations) from the soil and thus starve those competitors that require iron for their growth and survival, (iv) forming biofilms to increase competitiveness, (v) releasing H^+^ ions to acidify soil to create conditions unfavorable for their competitors, and (vi) direct predation or parasitism on their competitors.

Two groups of competition types exist: “exploitative competition” and “interference competition” (Glossary Box). Bacteria and fungi have developed various strategies to outcompete each other for C and energy resources in soil by both types and via niche partitioning (Fig. 5).

In “exploitative competition,” bacteria outcompete for easily available organic compounds (Fig. 5) for several reasons. The smaller size of bacterial cells results in a larger surface area-to-volume ratio than that of fungal cells, enabling bacteria to contact and take up small dissolved organic compounds faster (Table 2). The growth rate of bacteria is much faster (Table 2), enabling them to more rapidly occupy the organic matter resources. Finally, the cell membrane structure of bacteria is simpler than that of fungi, which facilitates direct uptake of small compounds from the soil solution [86].

In contrast, fungi outcompete for complex organic compounds (Fig. 5) mainly because they efficiently produce nearly all exoenzymes to decompose such structures [87]. Fungi can exploit more abundant C and energy sources than bacteria because their hyphae increase the habitat and exploration volume (Table 2), whereas unicellular bacteria are limited to a small volume in soil [56, 57]. The translocation of substances from remote locations by the mycelium [58] and slow growth (Table 2) help fungi to maintain a more stable state in soils, providing more time to decompose complex compounds than is available to bacteria [59].

In “interference competition,” both groups produce compounds to suppress or kill each other (Fig. 5). Bacteria can produce bacteriocins (a group of small antimicrobial peptides or proteins) [23, 88] and antifungal compounds [66] to reduce or even stop fungal growth. In turn, fungi can produce antibiotics (e.g. penicillin, streptomycin, and tetracycline produced by *Penicillium* and *Streptomyces*) [89] and mycotoxins to limit bacterial growth [90]. Volatile compounds produced by both groups diffuse through air-filled pores and suppress the activity and growth of competitors [91].

Both groups can form biofilms to compete for local C and energy sources by excluding the competitors (Fig. 5). Biofilms increase competitiveness by accumulating antagonist molecules due to the slow outward diffusion of compounds [92]. Biofilms also protect enclosed bacteria or fungi from predators and facilitate certain species to grow toward C-rich areas [93]. Quorum sensing is a common strategy adopted by many species to form biofilms by regulating the production of extracellular polymeric substances, surface attachment, motility, and dispersal [94, 95]. Through quorum sensing, the microbial populations can collectively respond to competition, for example, by ramping up antimicrobial compound production and by altering their behavior to gain a competitive advantage.

Both bacteria and fungi produce specific hydrolytic enzymes (e.g. chitinases, proteases) to break down the cell walls or proteins of their competitors [96]. Siderophores are produced by both groups to chelate iron from the soil and thus starve their competitors, which require iron for growth and survival [97].

Microorganisms can indirectly suppress each other by altering the physico-chemical properties of the environment (Fig. 5). For example, within the competitive interactions between *Collimonas fungivorans* and *Aspergillus niger*, the former produces acids to create conditions unfavorable for the latter [98]. Some representatives can act as predators or parasites on the other microbial group, leading to direct competition for survival. Mycophagy enables certain bacteria to predate living fungal hyphae to obtain nutrients and C [99]. For example, the number of *Collimonas*, a bacterial genus known for mycophagous growth, increased 4-fold over 2 weeks after invading *Absidia* hyphae in soil [100].

### Niche differentiation

**Figure 6:**
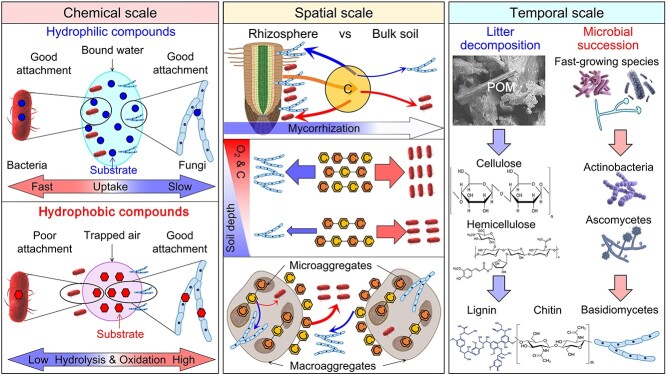
Niche differentiation between bacteria and fungi at chemical (left), spatial (middle), and temporal (right) scales. At the chemical scale, bacteria can rapidly take up hydrophilic organic compounds, thus outcompeting fungi. The latter outcompete the former for hydrophobic organic compounds because of their more effective exoenzymes and the better attachment of hyphae on hydrophobic surfaces. At the spatial scale, (i) fungi are more competitive in the rhizosphere than in bulk soil because rhizodeposition decreases the intensity of competition and the root colonization by mycorrhizal fungi, (ii) bacteria outcompete fungi for carbon with soil depth, and (iii) bacteria outcompete fungi in macroaggregates, whereas fungi compete better in microaggregates than in macroaggregates. At the temporal scale, microbial succession during plant litter decomposition follows the sequence: fast-growing microorganisms, followed by fungi and bacteria with cellulolytic, hemicellulolytic, and pectolytic abilities, then fungi that can decompose lignin and chitin.

Niche differentiation is the consequence of and a mechanism to decrease competitive interactions. Chemical, spatial, and temporal niche differentiation is distinguished here (Fig. 6).

#### Chemical niche differentiation

In terms of organic chemistry, bacteria outcompete fungi for hydrophilic compounds, while the latter are adapted to utilize hydrophobic compounds (Fig. 6). This is supported by up to 16 times greater incorporation of hydrophilic compounds into bacterial versus fungal phospholipids in upland and paddy soils after a 2-day soil incubation with ^13^C-labeled maize residues [70]. In contrast, the incorporation of hydrophobic compounds into fungal phospholipids was 1.5 times greater than that into bacterial phospholipids [70].

Bacteria outcompete for hydrophilic compounds mostly because of their fast growth and larger surface area-to-volume ratio (Table 2), which facilitates colonizing soil surfaces rich in hydrophilic compounds [71]. Organic compounds with larger NOSC values are more hydrophilic (i.e. more –COOH and = C=O groups) and thus easily available for microbial uptake from soil solution. Importantly, hydrophilic compounds have a high energy availability [43] because they are already soluble [no production of (per)oxidases is required] and their microbial uptake through cell membranes requires less energy than that for hydrophobic compounds [44, 72].

From the elemental stoichiometry perspective, bacteria have to outcompete fungi for hydrophilic N-rich compounds because the C:N ratio in bacterial biomass is two times lower than that in fungal biomass [19]. Generally, hydrophilic compounds (e.g. amino acids, peptides, amino sugars) have lower C:N ratios than hydrophobic compounds [31]. This is confirmed by the predominance of bacteria in the short-term assimilation of plant-derived N [101].

Fungi outcompete for hydrophobic compounds by relying on their broad and effective exoenzymes (Table 4) and the better attachment of hyphae on hydrophobic surfaces (Fig. 6). Complex hydrophobic compounds must be broken down and/or oxidized by extracellular enzymes outside the cell before they can be taken up. The first step in the depolymerization of most hydrophobic compounds is oxidation catalyzed by extracellular oxidative enzymes (e.g. peroxidases, phenol oxidases, laccases). The oxidation of hydrophobic compounds produces more –COOH, =C=O, and ≡C–OH groups, boosting their hydrophilicity. Although some bacteria can produce oxidative enzymes, their enzymatic activity and abundance are much lower than that of fungi (Table 4) [102]. Consequently, fungi dominate the decomposition of hydrophobic compounds.

**Table 4:** Characteristics of major cellulolytic and ligninolytic enzymes.

Enzymes	Sources	Substrates	References
Cellulase	Fungi and bacteria	Cellulose	[60]
β-Glucosidases	Fungi and bacteria	Cellulose	[60]
Endoglucanases	Fungi and bacteria	Oligosaccharides	[60]
Cellobiohydrolase	Fungi and bacteria	Cellobiose	[60]
Cellodextrinase	Fungi	Cellobiose	[60]
Xylanase	Fungi and bacteria	Xylan	[61]
Xyloglucanase	Fungi and bacteria	Xyloglucan	[61]
β-Xylosidase	Fungi and bacteria	Xylobiose, *p*-nitro-phenyl-β-D-pyranoside	[61]
Mannanase	Fungi and bacteria	Mannan, cellulose, xylan	[61]
Arabinanase	Fungi and bacteria	Arabinan	[61]
Laccase	Fungi and bacteria	Phenolics, aromatic amines	[62]
Lignin peroxidase	Fungi	Phenolics, aromatic amines, aromatic ethers, polycyclic aromatics	[62, 63]
Manganese peroxidase	Fungi	Phenolics	[62, 63]
Versatile peroxidase	Fungi	High-redox-potential aromatic compounds	[62, 63]
Dye-decolorizing peroxidase	Fungi and bacteria	Dye compounds, carotenoids, phenolics	[62]

Microorganisms must invest substantial resources (energy, C, N, P, S) to make the hydrophobic compounds (i.e. many –CH_3_ groups, aliphatics, aromatics) from plant litter and microbial necromass utilizable. This requires investing considerable energy in synthesizing and releasing exoenzymes. For example, the energy cost of synthesizing β-glucosidase during a 150-day vegetation period is 1.9–14 × 10^−6^ J g^−1^ soil, assuming that the β-glucosidase content is 2 ng g^−1^ soil, and that 7.1 mol adenosine triphosphate (ATP) is required to polymerize one-mole amino acids [43]. Under real soil conditions, microorganisms invest at least two orders of magnitude more energy to produce all the enzymes that are involved in splitting polymeric compounds than for β-glucosidase. Accordingly, exoenzyme production decreases the net energy gain from organic compounds. Exoenzymes, however, are beneficial only if their substrates are available in high concentration and/or the enzymes can effectively transform the energy stored in the substrates to microbial biomass to offset the energy costs for their production [103]. Hydrophobic compounds with lower NOSC values have higher Gibbs free energy (Fig. 1), and the EUE of fungi using various substrates is higher than that of bacteria (Table 2) [19]. This is an advantage when competing for hydrophobic compounds.

Microbial cells require contact with hydrophobic compounds to utilize them. Fungi secrete hydrophobins that subsequently form an amphiphilic protein film to facilitate the formation and movement of aerial hyphae [104]. The hydrophobin film enables hyphal attachment on hydrophobic surfaces of organic compounds (Fig. 6) [104]. Some fungi such as Candida, Aspergillus, Ustilago, and Trichosporon can efficiently produce surfactants (e.g. sophorose lipids, mannosylerythritol lipids) to reduce their cell surface tension and weaken the attachment of bacterial cells on hydrophobic surfaces [105]. These strategies help fungi to outcompete bacteria for hydrophobic organic compounds.

#### Spatial niche differentiation

Spatial heterogeneity (e.g. rhizosphere and detritusphere versus bulk soil, topsoil versus subsoil, macroaggregates versus microaggregates) (Table 3) of organic compounds leads to spatial niche differentiation (Fig. 6). Bacteria outcompete fungi for easily available C and energy sources in both the rhizosphere and bulk soils [76], but the competitiveness of fungi is higher in the rhizosphere than in bulk soil (Fig. 6). This is confirmed by a higher fungi-to-bacteria ratio in the former versus the latter [73, 74]. This is because various mycorrhiza types occupy root surfaces and play a crucial role especially in the rhizosphere, capturing exudates released by roots into the soil.

The rhizosphere mainly selects for copiotrophic bacteria relative to bulk soil [71, 106]. This is confirmed by the up to 7-fold higher proportion of copiotrophic bacteria in the rhizosphere [107]. Higher abundances of those bacteria in the rhizosphere simultaneously accelerate C depletion and reduce the competitiveness of oligotrophic bacteria for C relative to bulk soil [71]. Consequently, the relative competitiveness of fungi in the rhizosphere is higher than in bulk soil because they obtain energy directly from roots (Fig. 6). In contrast, fungi relying on their efficient exoenzyme systems outcompete bacteria for niches that are rich in complex compounds, such as the detritusphere, biopores, the O horizon, and animal cadavers (Table 3) [75, 77].

The decrease in the quantity of fresh plant-derived C and SOM with soil depth leads to niche differentiation between bacteria and fungi (Fig. 6). The energy content of SOM strongly decreases with depth (e.g. 630–1800 GJ ha^−1^ at 0–20-cm and 280–4100 GJ ha^−1^ at 20–100-cm soil depth) [43]. However, the energy content per unit of C increases with soil depth due to the accumulation of poorly degradable but energy-rich hydrophobic compounds (lignin derivates, fatty acids, lipids, etc.) with depth [43]. The proportion of Gram-positive bacteria, in contrast, increases with soil depth, while those of Gram-negative bacteria and fungi decrease [26, 78, 108], lowering the fungi-to-bacteria ratio [78, 79]. This is because compacted soil structure with smaller pores partly filled by water in deeper soils decreases the O_2_ concentration, which slows or stops fungal growth (aerobic organisms) [82]. Similarly, bacteria outcompete fungi in anoxic niches (Table 3). Fewer or no roots in deeper soils strongly reduce parasitism or symbiosis between plants and fungi [109]. These mechanisms consequently decrease fungal competitiveness for organic compounds with soil depth (Fig. 6; Table 3).

The heterogeneous distribution of C and energy sources in aggregates of various sizes also leads to the niche differentiation between bacteria and fungi (Fig. 6). Easily available organic compounds are typically more abundant in macro- than in microaggregates [80, 83], whereas persistent organic compounds are strongly protected by and within microaggregates [110] (Fig. 6). The strong association of organic matter to minerals in microaggregates limits microbial accessibility [111]. Accordingly, the competition for C and energy sources is much stronger in macroaggregates (Fig. 6). This is supported by up to 2-fold higher numbers of negative links between bacteria and fungi in such larger aggregates [112]. Up to 3.6-fold higher positive bacteria–bacteria interactions in macro- versus microaggregates enable bacteria to outcompete fungi there [81]. Fast-growing bacteria therefore dominate microbial communities in macroaggregates (Table 3). This is confirmed by the higher abundance of Proteobacteria and Bacteroidetes (mainly utilizing labile C) and lower abundances of Acidobacteria, Chloroflexi, and Verrucomicrobia (with an oligotrophic life strategy) in macro- than microaggregates [80].

#### Temporal niche differentiation

Temporal niche differentiation is strongly supported by the domination of bacteria in earlier stages of plant litter decomposition and of fungi in the later stages (Fig. 6) [70, 84]. The succession of dominant microorganisms with litter decomposition follows the sequence: initial colonization by fast-growing microorganisms (e.g. bacteria, sugar fungi such as Zygomycetes) that consume easily available C sources, followed by fungi (e.g. Ascomycetes) and bacteria (e.g. Actinobacteria) with cellulolytic, hemicellulolytic, and pectolytic abilities, then by Basidiomycetes that can decompose lignin and chitin (Fig. 6) [38]. The succession of microbial communities during the decomposition of compounds with complex structures (e.g. lignin, cellulose) also supports temporal niche differentiation (Fig. 6; Table 3). For example, bacterial and fungal communities remained stable in the first week after cellulose addition, whereby Gram-positive bacterial biomass decreased by 13% and that of Gram-negative bacteria and fungi increased by 12%–50% over 2 weeks of cellulose decomposition [85]. In contrast, opposite patterns were observed after 2 months of cellulose addition [85]. This agrees with the results that the proportion of fungal biomass in total microbial biomass increased over 40 days after cellulose addition and then decreased after cellulose exhaustion in grassland soil [84].

## Global change impacts

**Figure 7:**
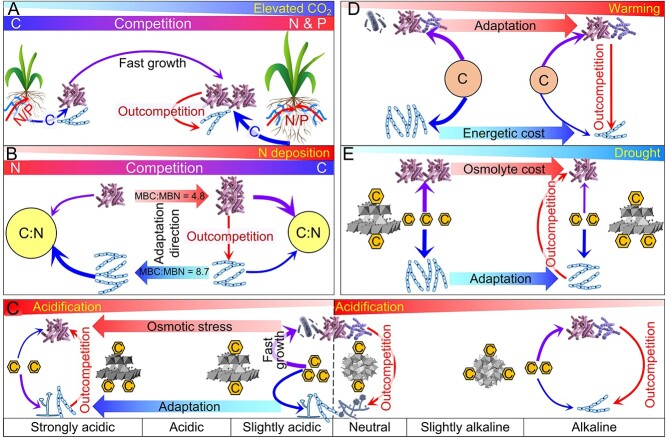
Global change impacts on bacterial–fungal competition for carbon (C) and energy sources. Effects of global changes include: elevated CO_2_ concentration in atmosphere (A), nitrogen (N) deposition (B), soil acidification (C), warming (D), and drought (E). The thickness of trapezoids, arrows, and lines indicates the relative intensities of the respective processes.

Within the broad range of global change components, five are especially relevant for processes in soil and may affect microbial communities as well as the competitiveness between bacteria and fungi for resources: elevated CO_2_, N deposition, soil acidification, global warming, and drought (Fig. 7). We also discussed the impacts of other regional climate changes (e.g. increased precipitation, permafrost thaw, increased fire frequency, land use change, salinization, and heavy metal contamination). Below, we describe the mechanisms of these effects on microbial communities, with a special focus on the competitive abilities of fungi and bacteria.

Understanding how global change processes interact with each other is crucial to predict bacterial–fungal interactions. Although limited studies have explored the effects of the suite of climate-associated changes that are expected in a given region or ecosystem on the soil microbial community and diversity [113–115], no general conclusions about bacterial–fungal competitive interactions to multifactor changes can be drawn at this point. We therefore avoid presenting potential interactive effects of these global change components because of the very high uncertainties involved. This highlights the great necessity to conduct long-term multifactor experiments to better understand the impacts of climate changes on bacterial–fungal interactions and their consequences for biogeochemical processes.

### Elevated CO_2_

The atmospheric CO_2_ concentration has steadily increased by >90 ppm over the past 50 years (https://www.co2.earth) and is expected to increase by an additional 300–600 ppm (RCP6.0 scenario) by 2100 [116]. Under elevated CO_2_ conditions, roots generally release more C, which increases microbial activities, especially in the rhizosphere [117, 118]. Consequently, increased C inputs under elevated CO_2_ stimulate the growth of r-strategists, mostly bacteria [117, 119]. Theoretically, increased root exudates will decrease the competition intensity between bacteria and fungi for C. Increased C availability, however, leads to a limitation of other nutrients, mostly N and P, for microbial growth (Fig. 7A) [118, 120]. This subsequently triggers strong competition for N and P between bacteria and fungi [121], which is especially intensive in nutrient-limited ecosystems (Fig. 7A). The effects of increased rhizodeposition under elevated CO_2_ conditions on competition therefore strongly depend on N and P availability.

Under the N- and P-rich conditions, bacteria quickly utilize the increased rhizodeposits from higher CO_2_ concentrations for their growth, boosting their biomass [122]. In contrast, increased rhizodeposition under N- and/or P-poor conditions stimulates microorganisms to decompose SOM to acquire more N and P, the so-called “priming effect” [117, 119, 123]. This favors exoenzyme-producing fungi to outcompete bacteria for recalcitrant organic C. A lower N availability for plants leads to a higher mycorrhization of roots under elevated CO_2_ concentrations, thus increasing the abundances of ecto- and arbuscular mycorrhizae communities [124]. Because bacterial demand for N is at least two times higher than that of fungi (the C:N ratio in bacterial biomass is 4.8, whereas that in fungal biomass is 8.7) [19], any N limitation more strongly affects bacteria than fungi, a condition that is especially expressed under elevated CO_2_ conditions. Therefore, elevated CO_2_ decreases the competition for C but increases the competition for N and the competitiveness of fungi because fungi have lower N demands (Fig. 7A) [125].

### Nitrogen deposition

Atmospheric N deposition has increased 3- to 5-fold over the 20th century [126]. Under N deposition, the competitiveness of bacteria for C and energy sources is expected to increase because of their higher N demands (Fig. 7B). This is confirmed by the decreased fungi-to-bacteria ratio under N deposition assessed by a meta-analysis across all terrestrial ecosystems [112]. A higher N availability with N deposition weakens the symbiosis between roots and ectomycorrhizal and arbuscular mycorrhizal fungi, thus decreasing the abundances of those fungi [127, 128].

Increased N deposition can result in two contrasting effects on bacteria, depending on the C availability. Increased N availability stimulates the growth of fast-growing Gram-negative bacteria (r-strategists) under C-rich conditions (e.g. increased rhizodeposition) [129, 130]. In contrast, Gram-positive bacteria will dominate microbial communities with the input of litter with a broader C/N ratio, the accumulation of recalcitrant C, and the reduction of C allocation belowground from plants after a long-term N deposition [112, 131]. In both scenarios, bacteria will outcompete fungi for C and energy sources (Fig. 7B). Nitrogen deposition, however, strongly accelerates soil acidification, which will have the opposite effects.

### Acidification

Soil pH crucially regulates microbial growth and competition because fungi and especially bacteria have their own (partly very narrow) optimal pH ranges. For example, the dynamics of the fungal:bacterial growth ratio at a pH range between 4 and 8 was a consequence of bacterial competitive suppression of fungal growth [6]. Therefore, soil acidification alters competitiveness for resources. The most relevant process causing soil acidification is N fertilization [e.g. the application of urea and especially (NH_4_)_2_SO_4_] [132].

Acidification of soils, especially those already acidic, boosts competition for C and energy sources for two reasons: (i) strongly reduced exoenzyme activities and/or substrate availability due to the sorption of enzymes and/or substrates on surfaces of sesquioxides [133] and (ii) increasing C and energy allocation to alleviate acidity stress (Fig. 7C) [134, 135]. For example, the pH drop from 4.5 to 3.8 resulted in up to 100 times higher ATP consumption by *Saccharomyces cerevisiae* without changes in ATP production [136]. Acidification raises the fungal competitiveness in acidic soils because fungi have much higher osmotic stress tolerance capabilities (Fig. 7C) [137], and because the optimal pH of exoenzymes produced by bacteria perform best at high (neutral to alkaline) pH, whereas fungal exoenzymes operate best at low (acidic) pH [138].

### Global warming

Temperature is one determinant for microbial metabolism [139], biomass [140], community composition [141], community succession [141], and interactions between species [142]. This is because microbial species differ greatly in their temperature-dependent adaptability and fitness in soil [143]. Notably, warming has many indirect effects as it modifies other factors (e.g. soil moisture, vegetation type and productivity, time) that play a role in selecting the microbial species. A long-term (up to 26 years) study suggested four general phases of SOM decomposition and associated microbial mechanisms with soil warming: rapid C loss through respiration; microbial community reorganization with lower fungal biomass and larger Gram-positive bacteria abundance after the depletion of labile C pools; a shift toward a more diverse, oligotrophic microbial community with lower fungal dominance and fungi/bacteria ratios; and a decrease of more recalcitrant C pools and microbial biomass [144].

Although warming reduces microbial biomass and diversity, the decrease of fungal biomass and diversity is larger than that of bacteria (Fig. 7D). For example, 7-year warming by +3°C decreased bacterial and fungal richness in grassland soils by 9.6% and 14.5%, respectively [145]. Warming increases bacterial dominance because fungi are more temperature sensitive than bacteria, whereas the temperature optimum (i.e. the temperature with the greatest activity) and the point of maximum temperature sensitivity (i.e. the point where the change in activity is greatest) is opposite [146]. This means bacteria acclimate better to warming (Fig. 7D). Bacterial communities change toward more thermophilic groups with warming, while fungal functional groups are unlikely to change. This is supported by increased populations and genes for labile-C decomposition, whereas the populations and genes for recalcitrant C decomposition remain stable under 2°C warming over 9 years [147]. Accordingly, bacteria are expected to be better competitors for C and energy sources with warming (Fig. 7D).

Warming may intensify competition [145, 148] because all microorganisms require more C and energy resources to meet their increased metabolic demands with increasing temperature [149]. The heat capacity (i.e. the amount of energy required to maintain a given amount of matter with increasing temperature by one °C) of fungi (−14 kJ mol^−1^ C °C^−1^) is three times larger than that of bacteria (−5 kJ mol^−1^ C °C^−1^) [146]. Accordingly, fungi must allocate more energy for biomass maintenance and growth. For example, assuming that the energy use efficiency of soil microorganisms using glucose as a substrate is 0.32 [19], and that microorganisms completely oxidize one mol glucose to 6 mol CO_2_ and yield 38 mol ATP (−30.5 kJ mol^−1^), then bacteria can convert 68 mmol more C into their biomass through the Calvin cycle (which requires 3 mol ATP for one mol C synthesis) [150] than fungi at a temperature increase of 1°C. Soil warming therefore weakens fungal competitiveness.

### Drought

Drought increases the intensity of competition for C and energy sources (Fig. 7E). Firstly, microorganisms must equilibrate to the osmotic conditions in soils by accumulating solutes (osmolytes) to retain water within their cells when the soil becomes drier [151]. Another strategy to alleviate drought stress is to produce extracellular polymeric substances (e.g. polysaccharides, proteins) [152], which can act as sponges to delay drying [151]. Osmolyte accumulation is an energetically expensive process and requires C [153], thus decreasing C and energy allocation for biomass maintenance and reproduction [154]. Secondly, drought may decrease C availability because of increasing sorption of organic compounds on metal oxides [155]. This can reach the solubility of products for ionic solutions and cause the collapse of repulsing charges of colloidal solutions. In contrast, up to nine orders of magnitude greater ionic strength under drought than under optimal water conditions may lead to C desorption from minerals [156]. Nevertheless, reduced soil moisture under drought strongly limits C diffusion [52], thus decreasing C accessibility for microorganisms, especially for bacteria. Thirdly, drought decreases the activities of various hydrolytic enzymes [157], thus lowering the SOM decomposition rate.

Fungi with a greater resistance to water limitation [52, 53] can outcompete bacteria for C and energy sources under drought (Fig. 7E). This is mainly because fungal hyphae can bridge spatially discrete resources [55] and because effective exoenzymes, especially oxidative enzymes, produced by fungi can decompose complex organic compounds [87].

### Other global change factors

Increased precipitation events will mainly occur in wet tropical and northern regions [116, 158]. Although increased precipitation has no direct effects on the fungi-to-bacteria ratio, bacteria may outcompete fungi for all resources as soil pores become water-filled and anaerobic with increasing moisture, especially when the initial soil moisture is low. Similarly, bacterial C and energy channels may dominate SOC dynamics in the Arctic as permafrost thaws [159] and fills the pores with water.

Although there are no consistent conclusions on the response of fungi and bacteria to heavy metal contamination, the former generally have a higher resistance [160, 161]. Accordingly, fungi may outcompete bacteria, especially with increasing contamination severity and duration.

Unlike the abovementioned global change factors, making generalizations about their impacts on bacterial–fungal competition for C and energy across ecosystems is challenging because these impacts depend on multiple factors and their interactions. For example, the changes in bacterial and fungal abundance depend on land use [162]. The increased area under cropland and tillage very strongly decreases microbial biomass but especially the fungal biomass, leading to overdominance of bacteria in agricultural soils [162]. The gradual change of water management in paddy soils—the reduced overflooding and water-saving technologies—lead to better soil aeration and a shift toward fungal communities as well as to Gram-positive bacteria.

The impact of increased wildfire frequency on this competition depends on fire severity and duration, soil resilience, and environmental conditions [163], whereby both groups are heat sensitive [164]. Nevertheless, the remaining persistent pyrogenic products on the surface and the increase of soil and organic matter hydrophobicity after wildfires will lead to a strong shift toward fungal communities [165].

Although fungi are commonly characterized by a stronger ability to cope with osmotic stress, the response patterns of both groups to salinization are habitat- or context-specific [166]. Salinization, especially in low-salinity soils, may accelerate the biomass loss of bacteria, which are less resistant to salt stress.

## Coexistence of bacteria and fungi

Soil microbial diversity is crucial in the functioning, stability, and health of terrestrial ecosystems. The stability of purely competitive or cooperative communities is weaker than that of communities with complex interactions [10, 167]. Although bacteria and fungi strongly compete for resources in soil, they always coexist in the same niches and have developed various cooperative interactions (e.g. cross-feeding, mutualism, commensalism). This coexistence helps both groups to increase the quality and quantity of substrates that they can feed on [24, 168] and to expand their spatial niche spaces [169, 170]. It also increases their resistance to environmental changes [18].

Metabolic dependencies are a major driver of this coexistence and of microbial community stability in general [171]. Although the two groups fundamentally compete for resources, the excreted products from one organism may be the preferred C, energy, and nutrient sources for another organism [170], leading to cross-feeding (Fig. 3). Such cross-feeding interactions are complex and pervasive in soil due to the great variability in feeding preferences (Fig. 4). The metabolites released by one population increase the quantity of substrates for another in cross-feeding.

Cross-fed bacteria and fungi are more resilient to environmental stress, especially nutrient limitation [130]. Fungi may benefit from the presence of bacteria, especially with respect to accessing organic N and removing growth inhibitors [172]. Basidiomycetes and arbuscular mycorrhiza lack efficient exoenzymes to access organic N directly [3]. In the mutual cross-feeding interactions between N-fixing bacteria and basidiomycetes, the bacteria continuously provide N to basidiomycetes and, in exchange, the bacteria utilize oligomers released by the fungal exoenzymes as C, energy, and other nutrients (e.g. P) [172]. The cross-feeding interactions are crucial in this coexistence, especially where one population consumes the toxic metabolites released by another and thus facilitates the growth of the metabolite-producing population [173].

The presence of fungi helps bacteria to expand niche space. This is confirmed by a 1.4-times-greater total expansion radius of *Pseudomonas aeruginosa* PAO1-*rfp* in the presence of hyphae of *Penicillium* sp. compared to without hyphae [169]. This is mainly because fungi create a micro-hydrophysical environment that increases bacterial motility and thus enables bacteria to colonize unoccupied niches [169]. Such positive effects strongly regulate bacterial diversity and functioning in soil. For example, the extraradical hyphae of arbuscular mycorrhizal fungi transport phosphate-solubilizing bacteria to organic P patches and thus accelerate organic P mineralization [174]. Such coexistence increases the resistance to allelochemical substances, thus alleviating the negative effects of such substances on population expansion. In the mutualism between *Burkholderia terrae* BS001 and nonwood decay fungi, *B. terrae* BS001 protects the fungi through sorption or detoxification effects from antagonistic agents (e.g. cycloheximide, metabolites from the antagonistic bacteria) [175]. In turn, the bacterium benefits by acquiring organic compounds and nutrients released by the hyphae [175].

The coexistence of complex species networks increases the resistance of microbial communities to environmental changes. For example, although microbial OTU numbers decreased by 12% under warming versus under the control, the resulting positive associations were 43% larger under warming than under the control [18]. This suggests that the main strategy adopted by the microbial community to acclimate to warming is boosting cooperative behaviors among the taxa [18].

## Conclusions and future perspectives

The bacterial–fungal competition for C, energy, and nutrients in soils is universal. This makes the consequences of such interactions and their impacts on biogeochemical processes crucial for soil functioning. We demonstrated that bacteria are 1.4–5 times more efficient in incorporating simple organic compounds as substrates, whereas fungi are 1.1–4.1 times more effective in utilizing complex and persistent compounds. This is mainly because bacteria more rapidly incorporate small organic compounds through simpler cell membrane structures. In contrast, fungi produce very efficient exoenzymes to decompose complex compounds, enabling them to slowly obtain C and energy during the decomposition of complex and persistent compounds.

The strong exploitative competition outlined above leads to chemical, spatial, and temporal niche differentiation. Specifically, bacteria outcompete fungi for hydrophilic compounds (chemical niche), dominate C utilization in bulk soil, deep soil, macroaggregates, small pores (spatial niches), and anoxic locations (chemical niches), as well as dominate the decomposition of plant litter at the early stage. In contrast, effective exoenzymes help fungi to outcompete bacteria for hydrophobic and persistent (e.g. lignin and chitin) compounds and for niches that are rich in complex compounds, such as the detritusphere, biopores, and the O horizon. Root colonization increases fungal competitiveness for C and energy sources in rhizosphere soil.

Our review opens the following important questions to be addressed in the future:

(i) What are the differences in the C and energy requirements and investments of bacteria and fungi that make them become successful competitors?

(ii) What are the mechanisms of niche differentiation and its consequences under increasing competition between bacteria and fungi?

(iii) What are the mechanisms and consequences of this competition for C, energy, and nutrients at spatial and temporal scales?

(iv) How do the shifts of various resources (e.g. by global change factors) modify the competitiveness of bacteria and fungi and the consequences for their functioning in soil?

## Supplementary Material

Supplementary_materials_wrae073
