# Supplementary material for: Mechanisms and implications of bacterial–fungal competition for soil resources
Source: ISME J. 2024 May 1;18(1):wrae073. doi: 10.1093/ismejo/wrae073 (PMC11104273; doi:10.1093/ismejo/wrae073)
Supplement: Supplementary_materials_wrae073 [file supplementary_materials_wrae073.docx]

**Supplementary materials**

Mechanisms and implications of bacterial-fungal competition for soil resources

Running title: Bacterial-fungal competition in soil

Chaoqun Wang^1,2,†^, Yakov Kuzyakov^3^

^1^ Biogeochemistry of Agroecosystems, University of Göttingen, Göttingen, Germany

^2^ Faculty of Land and Food Systems, The University of British Columbia, Vancouver, Canada

^3^ Department of Soil Science of Temperate Ecosystems, University of Göttingen, Göttingen, Germany

^†^ **Correspondence**: Chaoqun Wang ([chaoqun.wang@forst.uni-goettingen.de](mailto:chaoqun.wang@forst.uni-goettingen.de)), Biogeochemistry of Agroecosystems, University of Göttingen, Büsgenweg 2, Göttingen 37077, Germany

**Data sources**

To evaluate the preferences of C and energy sources, we collected peer-reviewed articles published from 1980–2023 through Google Scholar (http://scholar.google.com). The keywords were ‘carbon’ AND ‘isotope’ AND ‘phospholipid’. Articles were selected based on the following criteria: the PLFA contents of both bacteria and fungi were measured in the same soil at the same time; the ^13^C enrichment in fungal and bacterial PLFAs was presented; and the C content in fungal and bacterial PLFAs was reported. A total of 155 data pairs (addition versus control) from 18 articles (listed in Table S1) were included in the meta-analysis. Added substrates were classed into two groups: low-molecular-weight compounds such as glucose, acetic acid, and amino acids; and high-molecular-weight substances – a group of organic compounds with lower microbial availability such as plant residues, cellulose, and biochar.

**Preferences for carbon and energy sources**

To evaluate the preferences for C and energy sources, we designed Equation 3 to normalize ^13^C-enrichment in fungal relative to bacterial PLFAs (^13^C_Fungi_/^13^C_Bacteria_) to the biomass C content in fungi relative to bacteria:

$Microbial utilization preference (MUP)=\frac{\frac{{}^{13}{C_{Fungi}}}{{}^{13}{C_{Bacteria}}}}{\frac{{Total C}_{Fungi}}{{Total C}_{Bacteria}}}$ (3)

where ^13^C_Fungi_ and ^13^C_Bacteria_ are ^13^C enrichment in fungal and bacterial PLFAs, respectively; and (Total C_Fungi_) and (Total C_Bacteria_) are the C content in fungal and bacterial PLFAs, respectively. Higher MUP values indicate higher substrate-C assimilation by fungi versus bacteria. When MUP values exceed 1, fungi outcompete bacteria for the given substrate, and vice versa. The mean MUP values are 0.44 (ranging from 0.20 to 0.72) for low-molecular-weight compounds as substrates and 3.4 (ranging from 1.1 to 4.1) for high-molecular-weight compounds as substrates.

**Statistical analysis**

Power regression analysis was used to determine the relationships between normalized MUP using plant residues and incubation time (i.e. residue decomposition time) in Origin 2021 (OriginLab Corporation).

**Table S1.** List of references used for the meta-analysis

| **ID** | **Reference** | **Substrate type** | **Land use** |
| --- | --- | --- | --- |
| 1 | Apostel C, Herschbach J, Bore EK, Spielvogel S, Kuzyakov Y, Dippold MA. Food for microorganisms: position-specific ^13^C labeling and ^13^C-PLFA analysis reveals preferences for sorbed or necromass C. Geoderma. 2018;312:86‒94. | Low-molecular-weight compound | Cropland |
| 2 | Arao T. In situ detection of changes in soil bacterial and fungal activities by measuring ^13^C incorporation into soil phospholipid fatty acids from ^13^C acetate. Soil Biol Biochem. 1999;31(7):1015‒20. | Low-molecular-weight compound | Not reported |
| 3 | Arcand MM, Helgason BL, Lemke RL. Microbial crop residue decomposition dynamics in organic and conventionally managed soils. Appl Soil Ecol. 2016;107:347‒59. | High-molecular-weight substance | Cropland |
| 4 | Bai Z, Liang C, Bodé S, Huygens D, Boeckx P. Phospholipid 13C stable isotopic probing during decomposition of wheat residues. Appl Soil Ecol. 2016;98:65‒74. | High-molecular-weight substance | Cropland |
| 5 | Brant JB, Sulzman EW, Myrold DD. Microbial community utilization of added carbon substrates in response to long-term carbon input manipulation. Soil Biol Biochem. 2006;38(8):2219‒32. | Low-molecular-weight compound | Forest |
| 6 | Cao X, Zhang J, Yu Y, Ma Q, Kong Y, Pan W, et al. Alternate wetting–drying enhances soil nitrogen availability by altering organic nitrogen partitioning in rice-microbe system. Geoderma. 2022;424:115993. | Low-molecular-weight compound | Cropland |
| 7 | Dungait JAJ, Kemmitt SJ, Michallon L, Guo S, Wen Q, Brookes PC, et al. Variable responses of the soil microbial biomass to trace concentrations of ^13^C-labelled glucose, using ^13^C-PLFA analysis. Eur J Soil Sci. 2011;62(1):117‒26. | Low-molecular-weight compound | Grassland |
| 8 | Gunina A, Dippold M, Glaser B, Kuzyakov Y. Turnover of microbial groups and cell components in soil: ^13^C analysis of cellular biomarkers. Biogeosciences. 2017;14(2):271‒83. | Low-molecular-weight compound | Cropland |
| 9 | Heijboer A, De Ruiter PC, Bodelier PL, Kowalchuk GA. Modulation of litter decomposition by the soil microbial food web under influence of land use change. Front Microbiol. 2018;9:2860. | High-molecular-weight substance | Grassland |
| 10 | Herman DJ, Firestone MK, Nuccio E, Hodge A. Interactions between an arbuscular mycorrhizal fungus and a soil microbial community mediating litter decomposition. FEMS Microbiol Ecol. 2012;80(1):236‒47. | High-molecular-weight substance | Grassland |
| 11 | Koranda M, Kaiser C, Fuchslueger L, Kitzler B, Sessitsch A, Zechmeister-Boltenstern S, et al. Fungal and bacterial utilization of organic substrates depends on substrate complexity and N availability. FEMS Microbiol Ecol. 2014;87(1):142‒52. | Low-molecular-weight compound & High-molecular-weight substance | Forest |
| 12 | Lemanski K, Scheu S. Incorporation of 13C labelled glucose into soil microorganisms of grassland: effects of fertilizer addition and plant functional group composition. Soil Biol Biochem. 2014;69:38‒45. | Low-molecular-weight compound | Grassland |
| 13 | López-Mondéjar R, Brabcová V, Štursová M, Davidová A, Jansa J, Cajthaml T, et al. Decomposer food web in a deciduous forest shows high share of generalist microorganisms and importance of microbial biomass recycling. ISME J. 2018;12(7):1768‒78. | Low-molecular-weight compound & High-molecular-weight substance | Forest |
| 14 | Pan F, Li Y, Chapman SJ, Khan S, Yao H. Microbial utilization of rice straw and its derived biochar in a paddy soil. Sci Total Environ. 2016;559:15–23. | High-molecular-weight substance | Cropland |
| 15 | Phillips RL, Zak DR, Holmes WE, White DC. Microbial community composition and function beneath temperate trees exposed to elevated atmospheric carbon dioxide and ozone. Oecologia. 2002;131:236‒44. | Low-molecular-weight compound & High-molecular-weight substance | Forest |
| 16 | Rinnan R, Bååh E. Differential utilization of carbon substrates by bacteria and fungi in tundra soil. Appl Environ Microbiol. 2009;75(11):3611‒20. | Low-molecular-weight compound & High-molecular-weight substance | Tundra |
| 17 | Williams MA. Response of microbial communities to water stress in irrigated and drought-prone tallgrass prairie soils. Soil Biol Biochem. 2007;39(11):2750‒57. | Low-molecular-weight compound | Grassland |
| 18 | Zak DR, Kling GW. Microbial community composition and function across an arctic tundra landscape. Ecology. 2006;87(7):1659‒70. | Low-molecular-weight compound & High-molecular-weight substance | Tundra |
